# The Word Composite Effect Depends on Abstract Lexical Representations But Not Surface Features Like Case and Font

**DOI:** 10.3389/fpsyg.2017.01036

**Published:** 2017-06-20

**Authors:** Paulo Ventura, Tânia Fernandes, Isabel Leite, Vítor B. Almeida, Inês Casqueiro, Alan C.-N. Wong

**Affiliations:** ^1^Faculdade de Psicologia, Universidade de LisboaLisboa, Portugal; ^2^Departamento de Psicologia, Universidade de ÉvoraÉvora, Portugal; ^3^Department of Psychology, The Chinese University of Hong KongShatin, Hong Kong

**Keywords:** perceptual expertise, visual word recognition, holistic effect, composite task, alternating-case, handwritten forms

## Abstract

Prior studies have shown that words show a composite effect: When readers perform a same-different matching task on a target-part of a word, performance is affected by the irrelevant part, whose influence is severely reduced when the two parts are misaligned. However, the locus of this word composite effect is largely unknown. To enlighten it, in two experiments, Portuguese readers performed the composite task on letter strings: in Experiment 1, in written words varying in surface features (between-participants: courier, notera, alternating-cAsE), and in Experiment 2 in pseudowords. The word composite effect, signaled by a significant interaction between alignment of the two word parts and congruence between parts was found in the three conditions of Experiment 1, being unaffected by NoVeLtY of the configuration or by handwritten form. This effect seems to have a lexical locus, given that in Experiment 2 only the main effect of congruence between parts was significant and was not modulated by alignment. Indeed, the cross-experiment analysis showed that words presented stronger congruence effects than pseudowords only in the aligned condition, because when misaligned the whole lexical item configuration was disrupted. Therefore, the word composite effect strongly depends on abstract lexical representations, as it is unaffected by surface features and is specific to lexical items.

## Introduction

Written word recognition is a highly demanding visual task, whose acquisition has strong and visible impacts on all stages of visual processing (for a recent review, see, Dehaene et al., 2015). Indeed, learning to read leads to a general enhancement of early visual responses in the primary occipital cortex and to a better differentiated mosaic of category-specific regions in ventral visual cortex (Dehaene et al., 2010). Therefore, several authors have suggested that visual word recognition can be seen as a type of perceptual expertise, but one that differs from the face-like, subordinate-level expertise (McCandliss et al., 2003; Dehaene et al., 2005, 2015; Wong et al., 2009).

Indeed, in contrast to faces, visual word recognition seems to rely more on part-feature processing (e.g., Pelli et al., 2003). In order to identify a visual word, the identity, number, and order of elements are crucial and detailed spatial relationships among letters are uninformative (Grainger, 2008). In this vein, Farah (1991, 1992), Tanaka and Farah (1993), Farah et al. (1998) portrayed word and face perception as the two extremes of the continuum of visual object recognition: part-based processing for words vs. holistic processing for faces. This framework agrees with the observation that visual word and face processing are underpinned by selective neural mechanisms (e.g., Kanwisher et al., 1997; Cohen et al., 2000). On the other hand, one could argue that expert word perception involves at least certain aspects of holistic processing. For example, there is the well-known word superiority effect (Reicher, 1969; Wheeler, 1970) in which people have better recognition of letters presented within words as compared to isolated letters. These data suggest that whole word representation exist and can affect recognition at the letter or feature level (McClelland and Rumelhart, 1981; Rumelhart and McClelland, 1982).

**FIGURE 1:**
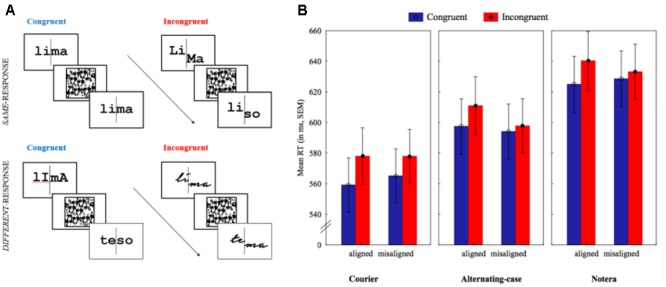
Illustration of the composite task with Portuguese words and the results of Experiment 1. **(A)** Sequence of events in the same-different matching composite task (Response × Alignment × Congruency) in Experiment 1. Font was manipulated between-participants (for details see General Method): courier (typical print font; illustrated in the same – congruent condition at the top); alternating-case (illustrated in same-incongruent and misaligned condition at the top and different congruent at the bottom) notera (simulating handwriting; in different-incongruent and aligned condition at the bottom). This is an illustration. In the experiment, we ensured that material was presented in the center of the screen.**(B)** Mean RTs (in ms) separately by font in the aligned and misaligned blocks with congruent trials in blue and incongruent trials in read. Error bars represent *SEM*.

In order to examine whether visual word recognition presents the signature of perceptual expertise, Wong et al. (2011, 2012) and Chen et al. (2013) have recently adopted a paradigm borrowed from the face-expertise literature, i.e., the *composite* task. In this task, observers are asked to decide whether a critical half of two sequential stimuli (e.g., the left half of words; for an illustration, see **Figure 1A**) is the same or not.

In the complete version of this task (for a recent meta-analysis and review see, Richler and Gauthier, 2014), the *response* (same vs. different) and *congruency* between the halves of the two stimuli (congruent vs. incongruent) are orthogonally manipulated (see **Figure 1A**). In *same – congruent* trials, the critical and the irrelevant half of the two stimuli are the same; in the *same* – *incongruent* trials, the critical halves of the two stimuli are the same but the irrelevant halves are different; in *different* – *congruent* trials, the halves (both the critical and the irrelevant) of the two stimuli are different; and in *different* – *incongruent* trials the critical halves are different but the irrelevant halves are identical. The *congruency* effect (i.e., better performance in the congruent than in the incongruent condition) reflects the impact of the irrelevant part on response to the critical part. This effect is estimated in *aligned* and *misaligned* conditions, with the latter used as a control, with the two halves of the stimulus misaligned. The rationale is that any interference from the irrelevant part in performance on the critical part indicates automatic and compulsory processing of all parts. Therefore, if the congruency effect is due to holistic processing of the whole stimulus (i.e., with an intact configuration), then it should be severely reduced (or virtually eliminated) in the misaligned condition on which stimulus configuration is disrupted. In other words, the *composite effect* corresponds to a stronger congruency effect in the aligned than in the misaligned condition, signaled by a significant interaction between alignment and congruency.

This was indeed the pattern of results found by Wong et al. (2011, 2012) for written words. Crucially, native English readers showed a stronger congruency effect for English words than readers with English as second language, specifically in the aligned condition (Wong et al., 2011), and Chinese readers showed larger holistic effects for Chinese words than non-words (Wong et al., 2012). From this piece of evidence, the word composite effect seems to be a marker of visual-word expertise in both alphabetic and non-alphabetic scripts. Nonetheless, the locus of this effect is largely unclear. To enlighten it, in the present study, in two experiments, Portuguese readers performed the same-different composite task on letter strings presented in aligned and misaligned blocks (cf. Wong et al., 2011; see **Figure 1A**).

In Experiment 1, we specifically investigated whether the word composite effect were affected by low-level visual aspects of stimuli, by presenting Portuguese disyllabic consonant-vowel-consonant-vowel (CV.CV) words in three *fonts* (between-participants): *courier, notera*, and *aLtErNaTiNg-case* courier. Courier is a typical printed font, but notera simulates handwriting, and both handwritten and alternating-case words differ from typical, printed words in geometrical structure and perceptual difficulty, hindering visual word recognition. We thus tested two alternative hypotheses.

On the one hand, the word composite effect could occur at an early, visual stage of processing, given that the word composite effect found in Chinese readers for one-kanji words relates to the electrophysiological P1 index, occurring 100–140 ms after stimulus onset. In this case, the composite effect would be affected by surface features. Specifically, this effect (signaled by a stronger congruency effect in the aligned than in the misaligned condition) would be stronger for notera than for courier words, given that letters in handwritten words are noisy, ambiguous, (although we acknowledge that letters in notera font may be less “noisy” or “ambiguous” than in handwriting: the letters in handwriting contain a lot of information irrelevant from letter identity and that it is hard to determine the identity of a letter sometimes) and their physical form is affected by neighboring letters due to additional continuity and stronger reliance on configural information than printed words (Barnhart and Goldinger, 2013). For alternating-case words, the composite effect would be smaller than for courier words, because the novelty of alternating-case disrupts attentionally mediated grouping of letters, hindering word recognition. Additionally, alternating-case disrupts the visual codes that are normally used in word recognition (e.g., Mayall, 2002). This will reduce the normal support for the appropriate lexical entry.

Alternatively, if the locus of the word composite effect depended on the operation of the *visual word form* (VWF) system, and hence, on abstract representations (Cohen et al., 2000, 2002; Dehaene et al., 2001, 2004, 2005; McCandliss et al., 2003; Dehaene and Cohen, 2011), the word composite effect would be immune to changes in surface features, and hence, the significant interaction between alignment and congruency would not be modulated by font.

In Experiment 2, to assess whether the composite effect involved abstract representations that were prelexically assembled, Portuguese readers performed the composite task on CV.CV pseudowords presented in a typical printed font (courier). If the composite effect occurred at an early, prelexical stage of processing, then the interaction between alignment and congruency would also be found for pseudowords. Alternatively, if it stems from lexical representations, then only the congruence effect would be found given that, regardless of disruption of the CV.CV configuration, due to automatic phonological recoding of the written stimuli, performance would be affected by the irrelevant part even in the misaligned condition.

Finally, as a stringent test of the locus of the composite effect for written stimuli, a cross-experiment analysis was conducted to examine whether the congruency effect would be stronger for words than pseudowords exclusively in the aligned condition. Note that the composite effect is larger for words than for pseudowords in English readers (Wong et al., 2011), and Chinese readers only present it for words but not for non-words (Wong et al., 2012). Thus, if the composite effect depended on access to abstract lexical representations, then the congruency effect for Portuguese words would be significantly larger than that for pseudowords but only when the configural representation is preserved (in the aligned condition).

## General Method

### Participants

All participants were native Portuguese readers with normal or corrected to normal vision and no known history of a reading disorder. They were undergraduate students of Psychology at Universidade de Lisboa, and participated voluntarily in exchange of course credits or a bookstore voucher.

In Experiment 1, 141 participants were randomly assigned to one font condition: courier (*n* = 49), notera (*n* = 46), and alternating-case (*n* = 46).

In Experiment 2, 43 participants performed the composite task on pseudowords.

This study followed the Declaration of Helsinki, the Portuguese deontological regulation for Psychology, and was approved by the Deontological Committee of Faculdade de Psicologia of Universidade de Lisboa. All participants provided informed consent.

### Material and Procedure

In Experiment 1, 24 sets of four CV.CV Portuguese words were used as stimuli (presented in the Supplemental Material). Each word was divided into a left and a right half as illustrated in **Figure 1A**: the left half was always the critical one. Within set, the left and right halves of each word were interchanged to create the four words resulting from the orthogonal manipulation of response (same; different) and congruency (congruent; incongruent). Each word appeared four times as study and test stimuli in the experimental trials.

For each word, three black-line stimuli with 20 point size (250 pixels × 150 pixels; 3.44° × 1.04° at a viewing distance of 90 cm) were prepared: courier, notera, and alternating-case courier. The left and right halves of each word were separated by a vertical line. As shown in **Figure 1A**, in the misaligned condition, the right half of the word (for both the study and the test words) was moved down by 100 pixels (3.44° × 1.66°; at a viewing distance of 90 cm). Stimuli were presented in the aligned and misaligned condition in different blocks.

Stimuli’s presentation and data collection were controlled by E-Prime 2.0^1^. The sequence of events in experimental trials is illustrated in **Figure 1A**: stimuli were presented on the center of a 17″ CRT monitor, first, a fixation cross (500 ms), followed by the study stimulus (400 ms), then a mask (1 s), followed by the test stimulus, which remained on the screen until response or for a maximum of 2.5 s. Participants were asked to perform as quickly and as accurately as possible a same-different judgment on the left part of the test stimulus by button presses (two buttons of a PST Serial Response Box). Before the experimental trials, they were first presented with four examples on paper, for which they received feedback on the correct response; next, they performed 16 computerized practice trials on different stimuli but with the same procedure as that on experimental trials.

Participants performed eight blocks of experimental trials (four aligned; four misaligned; block- and trial-order randomized), each block comprised six sets of words (96 trials). For the group exposed to alternating-case words, study and test word-pairs were of four types: (i) lowercase study-word and alternated uppercase-lowercase test-word (e.g., lima-TeMa; see **Figure 1A**); (ii) lowercase study-word and alternated lowercase-uppercase test-word (e.g., lima-tEmA; see **Figure 1A**), and vice versa, e.g., (iii) LiMa-tema, and (iv) lImA-tema, respectively. This group was presented with four orders of the eight blocks, each order comprising two types of study and test word pairs.

**FIGURE 2:**
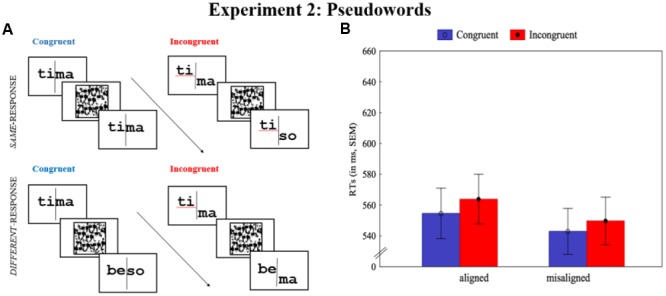
Illustration of the composite task with Portuguese pseudowords and the results of Experiment 2. **(A)** Aligned pseudowords (left-side) and misaligned pseudowords (right-side). **(B)** Mean RTs separately by alignment and congruency. Error bars represent SEM.

In Experiment 2, 24 sets of four CV.CV Portuguese pseudowords were created from the word sets used in Experiment 1 (and presented in the Supplemental Material). Whenever possible, the pseudowords were created by swapping the first consonant of one word from another within-set, or by changing the first consonant so that it resulted in a pseudoword. Material preparation (20 point size; courier font) and procedure were the same as in Experiment 1, as shown in **Figure 2A**; the only difference was that in the misaligned condition the distance between the left and right halves of each word was increased, resulting in a distance of 150 pixels (3.44° × 3.71°; at a viewing distance of 90 cm). The increase in misalignment (relative to that used on Experiment 1) was done to increase the chance of finding the crucial interaction between alignment and congruency.

## Results

**FIGURE 3:**
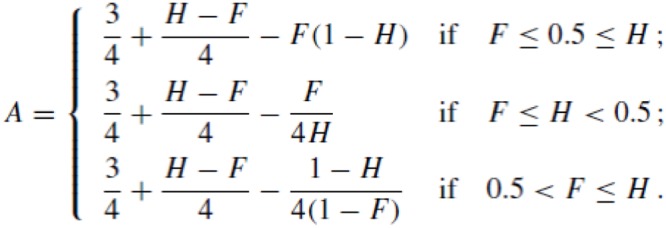
Formula used to compute the SDT *A* scores.

Due to excessive error rate (>25%), 12 participants were removed from the analyses in Experiment 1, and two participants in Experiment 2. The average global error rate for the other participants was below 3% in Experiment 1, and of ∼4% in Experiment 2. Data analyses were run, separately by experiment, on mean S*ignal Detection Theory* (SDT) *A* scores (Zhang and Mueller, 2005), with alignment (aligned; misaligned) and congruency (congruent; incongruent) as within-participants factors. *A* scores were computed as shown in **Figure 3**, with *hits* (H) corresponding to the proportion of correct responses in *different*-response trials and *false alarms* (F) to incorrect responses in *same*-response trials. Data analysis were also run on mean *Reaction Time* (RT) for correct responses (across response-type; after trimming of outliers 2.5 SD above or below the grand mean RT for each participant; <4% data excluded in each experiment). Effect sizes were analyzed according to partial eta square (η^2^) values (Cohen, 1988).

*A* was the SDT measure adopted because, in contrast to *d′*, it is a non-parametric measure of sensitivity that does not assume normality or equal variances (Zhang and Mueller, 2005), which was especially important because several participants had a hit rate of 1 in at least one congruent condition (39 in Experiment 1, and 11 in Experiment 2).

### Experiment 1

Participants were quite accurate on same-different judgments of the critical part of Portuguese words, with overall mean *A* scores of 0.98 (*SEM* = 0.002), in line with the results found previously with this task (cf. Wong et al., 2011, 2012).

In the mixed 3 (font: courier; notera; alternating-case) × 2 × 2 ANOVA, the main effect of font was significant, *F*(2,127) = 6.38, *p* < 0.0005, η^2^ = 0.09, *MSE* = 0.0007. Bonferroni corrected tests showed that participants had better performance when Portuguese words were presented in aLtErNaTiNg case (*M* = 0.99, *SEM* = 0.002) than when presented in handwritten notera (*p* = 0.001; *M* = 0.98, *SEM* = 0.002). No other differences were significant. The main effect of congruency was also significant, *F*(1,127) = 24.21, *p* < 0.0001, η^2^ = 0.16, *MSE* = 0.0002, with higher mean *A* scores in congruent (*M* = 0.99; *SEM* = 0.002) than in incongruent (*M* = 0.98; *SEM* = 0.002) trials. No other significant effects were found: main effect of alignment, *F*(1,127) = 1.28, *p* = 0.26, η^2^ = 0.01, *MSE* = 0.0002; all interactions, *F*s < 1. Due to the existence of large ceiling effects in *A*-values, which might have obscured the magnitude of any existing differences between the three experimental conditions and the detection of any interaction effects, data analysis were run on *RT*, an alternative outcome measure. As illustrated in **Figure 1B**, in the mixed ANOVA run on mean correct RTs, the critical interaction between alignment and congruency was highly significant, *F*(1,127) = 14.6, *p* < 0.0001, η^2^ = 0.10, *MSE* = 174. Indeed, the significant congruency effect found in the aligned condition (on average, participants were 16 ms faster, *SEM* = 1.9, in congruent than in incongruent trials), *F*(1,127) = 70.08, *p* < 0.0001, was significantly reduced in the misaligned condition (average congruency effect of 7 ms, *SEM* = 2.0), *F*(1,127) = 12.68, *p* < 0.001, and hence, the effect of congruency was significantly larger when Portuguese words were aligned than misaligned, *t*(130) = 3.82, *p* < 0.0005.

No significant main effect of alignment was found, *F* < 1, but the main effect of congruency was significant, *F*(1,127) = 54.14, *p* < 0.0001, η^2^ = 0.30, *MSE* = 317, and the main effect of font was marginally significant, *F*(2,127) = 2.99, *p* = 0.054, η^2^ = 0.04, *MSE* = 55167, because, as shown in **Figure 1B** and demonstrated in *post hoc* HSD Tukey tests, participants were overall faster when words were presented in typical courier than in handwritten case (*p* = 0.038; the other two comparisons, *p*s > 0.40).

Crucially, font did not modulate the word composite effect, not even remotely: Alignment × Congruency × Font, *F*(2,127) = 0.41, *p* = 0.67, η^2^ = 0.01, *MSE* = 174. In order to provide more single-degree-of-freedom more stringent tests to our hypotheses, we next examined the word composite effect separately for each font. As shown in **Figure 1B**, the interaction between alignment and congruency was significant for handwritten words, *F*(1,41) = 4.22, *p* = 0.046, η^2^ = 0.09, *MSE* = 287, alternating-case WoRdS, *F*(1,42) = 6.25, *p* = 0.016, η^2^ = 0.13, *MSE* = 167, and also for the typical, courier font, *F*(1,44) = 5.25, *p* = 0.027, η^2^ = 0.11, *MSE* = 76. Therefore, there was no significant impact of font (relative to typical printed font, neither a reduction of the congruency effect for mixed-case nor an increase of the congruency effect for notera font) in the word composite effect, suggesting that it stems from abstract, feature-independent representations.

### Experiment 2

In the ANOVA run on *A* scores for Portuguese pseudowords, the interaction between alignment and congruency was significant, *F*(1,41) = 143,4, *p* < 0.0001, η^2^ = 0.78, *MSE* = 0.015, but it was in the opposite direction than that signaling the composite effect. To put it differently, contrary to the composite effect, the effect of congruency was smaller in the aligned (*M* = 0.01, *SEM* = 0.002) than in the misaligned (*M* = 0.45, *SEM* = 0.002) condition. The two main effects were also significant: alignment, *F*(1,41) = 118.9, *p* < 0.0001, η^2^ = 0.74, *MSE* = 0.017, with better performance when pseudowords were presented aligned (*M* = 0.98, *SEM* = 0.007) than misaligned (*M* = 0.76, *SEM* = 0.03); and congruency, *F*(1,41) = 164.63, *p* < 0.0001, η^2^ = 0.80, *MSE* = 0.014, with higher sensitivity for congruent (*M* = 0.98, *SEM* = 0.005) than incongruent (*M* = 0.75, *SEM* = 0.03) trials.

In the ANOVA run on correct RTs, as shown in **Figure 2B**, both main effects were significant: alignment, *F*(1,41) = 7.56, *p* = 0.009, η^2^ = 0.16, *MSE* = 924; congruency, *F*(1,41) = 23.81, *p* < 0.0001, η^2^ = 0.37, *MSE* = 115. Most important, a composite effect was not observed, given that no significant interaction between alignment and congruency was found, *F* < 1. In other words, the disruption of stimulus’ configuration in the misaligned condition did not lead to a significant reduction of the congruency effect (*M* = 6.8, *SEM* = 2.4) relative to the aligned condition (*M* = 9.3, *SEM* = 2.4).

In this vein and considering the hypothesis that the word composite effect may result from access to abstract lexical representations, the direct comparison (unilateral *t*-test) between the congruency effect (computed on correct RTs) found for Portuguese words in Experiment 1 and for pseudowords in Experiment 2 revealed that the congruency effect was larger for Portuguese words but only in the aligned condition, *t*(170) = 1.85, *p* = 0.033, not in the misaligned, *t* < 1, on which stimulus’ configuration was disrupted.

## General Discussion

In the present study we examined the locus of the word composite effect (cf. Wong et al., 2011, 2012) by presenting Portuguese readers with written words and pseudowords in the composite task. Specifically, in Experiment 1, we investigated whether the word composite effect occurs at an early, low-level stage of visual processing, influenced by surface features (i.e., font: courier, notera, or alternating-cAsE). Indeed, electrophysiological evidence on Chinese readers recently suggested that the word composite effect could be affected by low-level visual features, given that for one-kanji words the effect modulated the P1 index, occurring 100–140 ms after stimulus onset (Chen et al., 2013). From this evidence, it could be the case that depending on font the composite effect (i.e., stronger congruency effect in the aligned condition) would be smaller for mixed-case words given the novelty of this configuration and disruption of the whole representation/the visual codes that are used in word recognition (e.g., Mayall et al., 1997; Mayall, 2002; Braet and Humphreys, 2006; Reingold et al., 2010) and larger for handwritten forms due to their stronger reliance in geometrical structure and configural information than printed words, Barnhart and Goldinger, 2013).

Much on the contrary, in Experiment 1, the word composite effect signaled by the significant alignment by congruency interaction was immune to font. Participants showed faster performance on congruent than incongruent trials especially in the aligned condition, and this congruency effect was significantly reduced when words were misaligned.

This pattern of results shows that this effect, originally reported in Chinese and English readers by Wong et al. (2011, 2012), is robust. Visual word recognition presents the signature of perceptual expertise: the automatic and compulsory processing of all parts of a word. Most important, the present results demonstrate that the word composite effect is immune to surface features of words: the same strong congruency effect, severely reduced in the misaligned condition, was found for handwritten and alternating-cAsE fonts as for a typical print font. These results agree with prior findings with the masked priming paradigm (which taps into early but already abstract stages of visual-word processing), demonstrating that both handwritten and alternating-case prime words are as effective as printed primes on word recognition (Gil-Lopez et al., 2011; Perea et al., 2015). In sum, the results of Experiment 1 demonstrate that the word composite effect stems from access to abstract representations. The multiple encounters with words through reading experience give rise to a perceptual system that is able to neglect visual differences that do not alter what is invariant in a word – the identity and sequence of letters (Dehaene, 2009).

At what stage of processing does the holistic processing of words take place? The VWFA is generally agreed to intervene in the efficient identification of orthographic stimuli (Dehaene et al., 2001) and to enable quick association of such stimuli with phonological and lexical information (Hashimoto and Sakai, 2004). The VWFA contains specialized neuronal circuitry for orthographic coding (Dehaene et al., 2015): with alphabetic expertise, the VWFA develops an efficient bottom-up hierarchy of tuned cells for letters, bigrams, morphemes and short words. Indeed, in expert alphabetic readers, the VWFA is organized in a posterior-to-anterior hierarchy (Dehaene et al., 2004; Vinckier et al., 2007; Thesen et al., 2012): posterior parts respond to individual letters, irrespective of case (Dehaene et al., 2004; Thesen et al., 2012), whereas anterior parts respond to letter combinations such as bigrams (Binder et al., 2006; Vinckier et al., 2007). An fMRI study using repetition suppression even suggests that some neurons in the VWFA may sharply tune to known words (Glezer et al., 2009; Dehaene et al., 2015). Holistic processing may intervene to bind together individual letters that activate the posterior part of the VWFA providing the input that activates more anterior parts of the VWFA, responsive to whole words.

The composite effect was not observed for Portuguese pseudowords. We observed a congruence effect for pseudowords, given that, regardless of disruption of the CV.CV configuration, due to automatic phonological recoding of the written stimuli, performance was affected by the irrelevant part even in the misaligned condition. If anything, the composite effect for pseudowords was in the opposite direction than that signaling the usual composite effect.

The congruency effect was significantly larger for Portuguese words than for pseudowords but only in the aligned condition, suggesting that more than depending on access to abstract orthographic representations, the composite effect has a lexical locus.

It could be argued that instead of an involvement of the VWF system, attentional spatial demands to multiple elements could underpin the word composite effect. Indeed, all parts of a word are diagnostic to its identity, and word recognition relies on efficient mechanisms of letter identity and letter position (Grainger, 2008). Therefore, attentional mechanisms, supported by the *posterior parietal cortex* (PPC; Reilhac et al., 2013), could instead be responsible for holistic processing of words. This would agree with the results of Chua et al. (2015), who recently showed that for non-face objects of expertise, after laboratory training, the emergence of the composite effect depended on spatial attention. However, this possibility seems unlikely, taking into account the results obtained in Experiment 1 with alternating-case words and those obtained in Experiment 2 with pseudowords.

Specifically, in Experiment 1, we found the same word composite effect for courier (typical printed) and alternating-case fonts. Although earlier studies suggested that alternating-case disrupts low-level early visual encoding of letters (e.g., Besner and Johnston, 1989), recent behavioral, neuropsychological, and neuroimaging studies have shown that the impact of alternating-case primarily influences attentional processes in the PPC and not early processes supported by occipital regions (e.g., Mayall et al., 1997; Braet and Humphreys, 2006; Braet and Humphreys, 2007; Reingold et al., 2010). Note that the PPC is the neural region that underpins visual spatial attention mediating multiple element processing including that of letter-strings (e.g., Lobier et al., 2012; Reilhac et al., 2013). Thus, if the locus of the word composite effect resulted from attentional demands of multiple-letter processing, given that case alternation specifically affects this attentional PPC mechanism, then we should have found a smaller congruency effect in the aligned condition for alternating-case words than for printed words. Much on the contrary, the congruency effect was virtually the same in both conditions, suggesting that it is not attention to multiple letters that is the locus of the composite effect. Furthermore, in Experiment 2, no such composite effect was found for pseudowords, and hence, this effect seems to depend on internal, abstract, and lexical representations, presumably supported by the VWF system.

It might seem at odds that surface features do not affect holistic processing of words, given the prior evidence with Chinese readers showed that the kanji-word composite effect was modulated at the electrophysiological P1 time-window (Chen et al., 2013). Note, however, that the present results were obtained with alphabetic readers, for whom no study to date has examined the neural underpinnings of the word composite effect. Additionally, it might be the case that feedback from more anterior, high-level areas (including the VWF area, and possibly those responsible for phonological or semantic processing) were involved in the electrophysiological results reported by Chen et al. (2013). Another possible reason for the apparent disparate results may reside in the nature of the actual manipulations carried out in the two studies. In Experiment 1, we tested the influence of visual differences that do not affect the identity of the letters, nor the sequence of letters in the word, on the word composite effect. On the opposite the kanji-word composite effect that was modulated at the electrophysiological P1 time-window in Chen et al. (2013) experiment resulted from misalignment of two parts of a character which may have interfered/impared the recognition of what has been learned to be a perceptual unit through reading experience. It thus may be the case that the manipulations conducted in the two studies are targeting two different mechanisms. Note that our word recognition system meets two apparent contradictory requirements: on one hand it is able to ignore significant visual differences, those that are processed as irrelevant to the identity of letters or to identify the sequence of letters, but it is highly sensible to changes that may hamper the identity or categorization of learned perceptual units (Dehaene, 2009). Further studies should thus examine these distinct possibilities.

The present results suggest that the word composite effect has a lexical orthographic locus, but phonological mediation could also be involved. Although the phonological code lags slightly beyond the orthographic code, both are rapidly activated (e.g., Van Orden, 1987; Ferrand and Grainger, 1993; Ziegler et al., 2000; Grainger et al., 2006; cf. Van Orden and Kloos, 2005 for a revision), and hence, the word composite effect could be modulated by *orthography-to-phonology consistency*. Note that, in Portuguese, an orthographic syllable (e.g.,<*ca*> in <cano> and <cave>; English translation: pipe and basement) can map into more than one phonological syllable (e.g., /k6/ in /k6nu/, and /ka/ in /kavə, respectively). Therefore, if phonology modulated the word composite effect (i.e., the magnitude of the congruency effect in the aligned condition relative to the misaligned one), then the congruency effect for orthography-to-phonology *inconsistent* words (e.g., cano; cave) would be significantly smaller than for *consistent* words (e.g., tive; tino; /tivə/ and /tinu/; English translation: (I) had; sense, respectively), and exclusively in the aligned condition. Future studies should examine this possibility.

In sum, the present results demonstrate that the word composite effect stems from access to abstract lexical representations, presumably underpinned by the VWF system. This effect is immune to changes in surface features and was not found for pseudowords.

## Author Contributions

PV, TF, and IL designed the experiments. PV, VA, and IC performed the experiments. PV and TF analyzed the data and drafted the manuscript. All co-authors provided critical reviews of prior versions of the manuscript.

## Conflict of Interest Statement

The authors declare that the research was conducted in the absence of any commercial or financial relationships that could be construed as a potential conflict of interest.

## Footnotes

1 www.pstnet.com/eprime.

## References

[B1] Barnhart A. S. Goldinger S. D. (2013). Rotation reveals the importance of configural cues in handwritten word perception. *Psychon. Bull. Rev.* 20 1319–1326. 10.3758/s13423-013-0435-y 23589201 PMC3748233

[B2] Besner D. Johnston J. C. (1989). *Reading and the Mental Lexicon: On the Uptake of Visual Information Lexical Representation and Process*. Cambridge, MA: MIT Press 291–316.

[B3] Binder J. R. Medler D. A. Westbury C. F. Liebenthal E. Buchanan L. (2006). Tuning of the human left fusiform gyrus to sublexical orthographic structure. *Neuroimage* 33 739–748. 10.1016/j.neuroimage.2006.06.053 16956773 PMC1634933

[B4] Braet W. Humphreys G. (2006). The ’special effect’ of case mixing on word identification: neuropsychological and transcranial magnetic stimulation studies dissociating case mixing from contrast reduction. *J. Cogn. Neurosci.* 18 1666–1675. 10.1162/jocn.2006.18.10.1666 17014372

[B5] Braet W. Humphreys G. (2007). A selective effect of parietal damage on letter identification in mixed case words. *Neuropsychologia* 45 2226–2233. 10.1016/j.neuropsychologia.2007.02.016 17382976

[B6] Chen H. Bukach C. M. Wong A. C.-N. (2013). Early electrophysiological basis of experience-associated holistic processing of chinese characters. *PLoS ONE* 8:e61221 10.1371/journal.pone.0061221 PMC3623809 23593436

[B7] Chua K.-W. Richler J. J. Gauthier I. (2015). Holistic processing from learned attention to parts. *J. Exp. Psychol. Gen.* 144 723–729. 10.1037/xge0000063 25775049 PMC4922746

[B8] Cohen J. (1988). *Statistical Power Analysis for the Behavioral Sciences* 2nd Edn. Hillsdale, NJ: Erlbaum.

[B9] Cohen L. Dehaene S. Naccache L. Lehericy S. Dehaene-Lambertz G. Henaff M. A. (2000). The visual word form area: spatial and temporal characterization of an initial stage of reading in normal subjects and posterior split-brain patients. *Brain* 123 291–307. 10.1093/brain/123.2.291 10648437

[B10] Cohen L. Lehericy S. Chochon F. Lemer C. Rivaud S. Dehaene S. (2002). Language-specific tuning of visual cortex? functional properties of the visual word form area. *Brain* 125 1054–1069. 10.1093/brain/awf094 11960895

[B11] Dehaene S. (2009). *Les Neurones de La Lecture.* Paris: Odile Jacob.

[B12] Dehaene S. Cohen L. (2011). The unique role of the visual word form area in reading. *Trends Cogn. Sci.* 15 254–262. 10.1016/j.tics.2011.04.003 21592844

[B13] Dehaene S. Cohen L. Morais J. Kolinsky R. (2015). Illiterate to literate: behavioural and cerebral changes induced by reading acquisition. *Nat. Rev. Neurosci.* 16 234–244. 10.1038/nrn3924 25783611

[B14] Dehaene S. Cohen L. Sigman M. Vinckier F. (2005). The neural code for written words: a proposal. *Trends Cogn. Sci.* 9 335–341. 10.1016/j.tics.2005.05.004 15951224

[B15] Dehaene S. Jobert A. Naccache L. Ciuciu P. Poline J. B. Le Bihan D. (2004). Letter binding and invariant recognition of masked words: behavioral and neuroimaging evidence. *Psychol. Sci.* 15 307–313. 10.1111/j.0956-7976.2004.00674.x 15102139

[B16] Dehaene S. Naccache L. Cohen L. Bihan D. LeMangin J.-F. Poline J.-B. (2001). Cerebral mechanisms of word masking and unconscious repetition priming. *Nat. Rev. Neurosci.* 4 752. 10.1038/89551 11426233

[B17] Dehaene S. Pegado F. Braga L. W. Ventura P. Nunes G. Jobert A. (2010). How learning to read changes the cortical networks for vision and language. *Science* 330 1359–1364. 10.1126/science.1194140 21071632

[B18] Farah M. J. (1991). Patterns of cooccurrence among the associate agnosias: implications for visual object representation. *Cogn. Neuropsychol.* 8 1–19. 10.1080/02643299108253364

[B19] Farah M. J. (1992). Agnosia. *Curr. Opin. Neurol.* 2 162–164. 10.1016/0959-4388(92)90005-6 1638147

[B20] Farah M. J. Wilson K. D. Drain M. Tanaka J. N. (1998). What is “special” about face perception? *Psychol. Rev.* 105 482–498. 9697428 10.1037/0033-295x.105.3.482

[B21] Ferrand L. Grainger J. (1993). The time course of orthographic and phonological code activation in the early phases of visual word recognition. *Bull. Psychon. Soc.* 31 119–122. 10.1111/j.1467-9280.2006.01821.x

[B22] Gil-Lopez C. Perea M. Moret-Tatay C. Carreiras M. (2011). Can masked priming effects be obtained with handwritten words? *Atten. Percept. Psychophys.* 73 1643–1649. 10.3758/s13414-011-0174-y 21751051

[B23] Glezer L. S. Jiang X. Riesenhuber M. (2009). Evidence for highly selective neuronal tuning to whole words in the “visual word form area. *Neuron* 62 199–204. 10.1016/j.neuron.2009.03.017 19409265 PMC2706007

[B24] Grainger J. (2008). Cracking the orthographic code: an introduction. *Lang. Cogn. Process.* 23 1–35. 10.1080/01690960701578013

[B25] Grainger J. Kiyonaga K. Holcomb P. J. (2006). The time-course of orthographic and phonological code activation. *Psychol. Sci.* 17 1021–1026. 10.1111/j.1467-9280.2006.01821.x 17201781 PMC1857302

[B26] Hashimoto R. Sakai K. L. (2004). Learning letters in adulthood: direct visualization of cortical plasticity for forming a new link between orthography and phonology. *Neuron* 42 311–322. 10.1016/S0896-6273(04)00196-5 15091345

[B27] Kanwisher N. McDermott J. Chun M. M. (1997). The fusiform face area: a module in human extrastriate cortex specialized for face perception. *J. Neurosci.* 17 4302–4311. 9151747 10.1523/JNEUROSCI.17-11-04302.1997 PMC6573547

[B28] Lobier M. Peyrin C. Le Bas J. F. Valdois S. (2012). Pre-orthographic character string processing and parietal cortex: a role for visual attention in reading? *Neuropsychologia* 50 2195–2204. 10.1016/j.neuropsychologia.2012.05.023 22659111

[B29] Mayall K. (2002). Case-mixing effects on children’s word recognition: lexical feedback and development. *Q. J. Exp. Psychol. A* 55 525–542. 10.1080/02724980143000334 12047057

[B30] Mayall K. Humphreys G. W. Olson A. (1997). Disruption to word or letter processing? The origins of case-mixing effects. *J. Exp. Psychol. Learn. Mem. Cogn.* 23 1275–1286. 10.1037/0278-7393.23.5.1275 9293635

[B31] McCandliss B. D. Cohen L. Dehaene S. (2003). The visual word form area: expertise for reading in the fusiform gyrus. *Trends Cogn. Sci.* 7 293–299. 10.1016/s1364-6613(03)00134-7 12860187

[B32] McClelland J. L. Rumelhart D. L. (1981). An interactive activation model of context effects in letter perception. Part 1. An account of basic findings. *Psychol. Rev.* 88 375–407. 10.1037/0033-295X.88.5.375 7058229

[B33] Pelli D. G. Farell B. Moore D. C. (2003). The remarkable infefficiency of word recognition. *Nature* 423 752–756. 10.1038/nature01516 12802334

[B34] Perea M. Vergara-Martínez M. Gomez P. (2015). Resolving the locus of cAsE aLtErNaTiOn effects in visual word recognition: evidence from masked priming. *Cognition* 142 39–43. 10.1016/j.cognition.2015.05.007 26010560

[B35] Reicher G. M. (1969). Perceptual recognition as a function of the meaningfulness of the stimulus material. *J. Exp. Psychol.* 81 275–280. 10.1037/h0027768 5811803

[B36] Reilhac C. Peyrin C. Démonet J.-F. Valdois S. (2013). Role of the superior parietal lobules in letter-identity processing within strings: FMRI evidence from skilled and dyslexicreaders. *Neuropsychologia* 51 601–612. 10.1016/j.neuropsychologia.2012.12.010 23270676

[B37] Reingold E. M. Yang J. Rayner K. (2010). The time course of word frequency and case alternation effects on fixation times in reading: evidence for lexical control of eye movements. *J. Exp. Psychol. Hum. Percept. Perform.* 36 1677–1683. 10.1037/a0019959 20731513

[B38] Richler J. J. Gauthier I. (2014). A meta-analysis and review of holistic face processing. *Psychol. Bull.* 140 1281–1302. 10.1037/a0037004 24956123 PMC4152424

[B39] Rumelhart D. E. McClelland J. L. (1982). An interactive activation model of context effects in letter perception: Part 2. The contextual enhancement effect and some tests and extensions of the model. *Psychol. Rev.* 89 60–94. 10.1037/0033-295X.89.1.60 7058229

[B40] Tanaka J. W. Farah M. J. (1993). Parts and wholes in face recognition. *Q. J. Exp. Psychol. A* 46 225–245. 10.1080/14640749308401045 8316637

[B41] Thesen T. McDonald C. R. Carlson C. Doyle W. Cash S. Sherfey J. (2012). Sequential then interactive processing of letters and words in the left fusiform gyrus. *Nat. Commun.* 3 1284 10.1038/ncomms2220 PMC4407686 23250414

[B42] Van Orden G. C. (1987). A ROWS is a ROSE: spelling, sound and reading. *Mem. Cognit.* 15 181–198. 10.3758/BF03197716 3600258

[B43] Van Orden G. C. Kloos H. (2005). “The question of phonology and reading,” in *The Science of Reading: A Handbook* eds Snowling M. S. Hulme C. (London: Blackwell Publishing) 61–78. 10.1002/9780470757642.ch4

[B44] Vinckier F. Dehaene S. Jobert A. Dubus J. P. Sigman M. Cohen L. (2007). Hierarchical coding of letter strings in the ventral stream: dissecting the inner organization of the visual word-form system. *Neuron* 55 143–156. 10.1016/j.neuron.2007.05.031 17610823

[B45] Wheeler D. D. (1970). Processes in word recognition. *Cogn. Psychol.* 1 59–85. 10.1016/0010-0285(70)90005-8

[B46] Wong A. C.-N. Bukach C. M. Hsiao J. Greenspon E. Ahern E. Duan Y. (2012). Holistic processing as a hallmark of perceptual expertise for nonface categories including Chinese characters. *J. Vis.* 12 7 10.1167/12.13.7 23220578

[B47] Wong A. C.-N. Bukach C. M. Yuen C. Yang L. Leung S. Greenspon E. (2011). Holistic processing of words modulated by reading experience. *PLoS ONE* 6:e20753 10.1371/journal.pone.0020753 PMC3116835 21698240

[B48] Wong A. C.-N. Palmeri T. J. Rogers B. P. Gore J. C. Gauthier I. (2009). Beyond shape: how you learn about objects affects how they are represented in visual cortex. *PLoS ONE* 4:e8405 10.1371/journal.pone.0008405 PMC2794531 20027229

[B49] Zhang J. Mueller S. T. (2005). A note on ROC analysis and non-parametric estimate of sensitivity. *Psychometrika* 70 203–212. 10.1007/s11336-003-1119-8

[B50] Ziegler J. C. Ferrand L. Jacobs A. Rey A. Grainger J. (2000). Visual and phonological codes in letter and word recognition: evidence from incremental priming. *Q. J. Exp. Psychol. A* 53 671–692. 10.1080/713755906 10994225

