# Supplementary material for: The Word Composite Effect Depends on Abstract Lexical Representations But Not Surface Features Like Case and Font
Source: Front Psychol. 2017 Jun 20;8:1036. doi: 10.3389/fpsyg.2017.01036 (PMC5476921; doi:10.3389/fpsyg.2017.01036)
Supplement: Supplementary file 1 [file Data_Sheet_1.docx]

*Supplemental material*

The twenty-four sets of four bisyllabic four-letter Portuguese words used to create the stimuli for the different experimental conditions (Experiment 1).

| Set | Words | | | |
| --- | --- | --- | --- | --- |
| 1 | BIFE (steak) | BICO (beak) | SAFE (get way) | SACO (bag) |
| 2 | BODE (goat) | BOGA (bogue) | RUDE (rude) | RUGA (wrinkle) |
| 3 | BULE (teapot) | BUDA (buddha) | ROLE (roll) | RODA (wheel) |
| 4 | DOSE (dose) | DOCA (dock) | BISE (encore) | BICA (spout) |
| 5 | DURE (last) | DUNA (dune) | MIRE (aim) | MINA (mine) |
| 6 | FIGO (fig) | FITA (ribbon) | NEGO (I deny) | NETA (granddaughter) |
| 7 | LISA (smooth) | LIGO (care) | PESA (weighs) | PEGO (I catch) |
| 8 | MEGA (mega) | MERO (mere) | FIGA (fig) | FIRO (I hurt) |
| 9 | PUDE (I could) | PUXA (pull) | LIDE (deal) | LIXA (sandpaper) |
| 10 | RIJA (tough) | RIME (rime) | FUME (smoke) | FUJA (run away) |
| 11 | SINA (lot) | SIGO (following) | RENA (reindeer) | REGO (gully) |
| 12 | VICE (vice) | VIGA (beam) | ROCE (rook) | ROGA (entreats) |
| 13 | BASE (base) | BAFO (breath) | PISE (tread) | PIFO (drunkness) |
| 14 | CUME (top) | CUJA (whose) | SOME (add) | SOJA (soy) |
| 15 | DIGA (say) | DITO (said) | NEGA (denies) | NETO (grandson) |
| 16 | FASE (phase) | FARO (flair) | VISE (aim) | VIRO (turn) |
| 17 | FOLE (bellows) | FORA (outside) | PULE (jump) | PURA (pure) |
| 18 | LEVA (takes) | LEGO (lego) | DIVA (diva) | DIGO (I say) |
| 19 | LIMA (lime) | LISO (smooth) | TEMA (theme) | TESO (stiff) |
| 20 | LUTE (fight) | LUVA (glove) | NOTE (notice) | NOVA (new) |
| 21 | REGA (irrigation) | REMO (rowing) | LIGA (alloy) | LIMO (slime) |
| 22 | TINA (tub) | TIPO (type) | CENA (scene) | CEPO (block) |
| 23 | TIVE (had) | TIRA (strip) | NOVE (nine) | NORA (daughter-in-law) |
| 24 | VIME (rattan) | VILA (town) | COME (eat) | COLA (glue) |

The twenty-four sets of four bisyllabic four-letter Portuguese pseudowords used to create the stimuli for the different experimental conditions (Experiment 2).

| Set |  | | | |
| --- | --- | --- | --- | --- |
| 1 | SIFE | SICO | DAFE | DACO |
| 2 | SODE | SOGA | BUDE | BUGA |
| 3 | RULE | RUDA | JOLE | JODA |
| 4 | JOSE | JOCA | NISE | NICA |
| 5 | SURE | SUNA | ZIRE | ZINA |
| 6 | NIGO | NITA | FEGO | FETA |
| 7 | JISA | JIGO | VESA | VEGO |
| 8 | FEGA | FERO | BIGA | BIRO |
| 9 | LUDE | LUXA | CIDE | CIXA |
| 10 | FIJA | FIME | TUME | TUJA |
| 11 | RINA | RIGO | BENA | BEGO |
| 12 | JICE | JIGA | LOCE | LOGA |
| 13 | LASE | LAFO | DISE | DIFO |
| 14 | VUME | VUJA | POME | POJA |
| 15 | ZIGA | ZITO | DEGA | DETO |
| 16 | TASE | TARO | LISE | LIRO |
| 17 | POLE | PORA | GULE | GURA |
| 18 | MEVA | MEGO | TIVA | TIGO |
| 19 | TIMA | TISO | BEMA | BESO |
| 20 | DUTE | DUVA | FOTE | FOVA |
| 21 | FEGA | FEMO | RIGA | RIMO |
| 22 | DINA | DIPO | TENA | TEPO |
| 23 | NIVE | NIRA | TOVE | TORA |
| 24 | DIME | DILA | VOME | VOLA |
